# A Combined XRD, Solvatochromic, and Cyclic Voltammetric Study of Poly (3,4-Ethylenedioxythiophene) Doped with Sulfonated Polyarylethersulfones: Towards New Conducting Polymers

**DOI:** 10.3390/polym10070770

**Published:** 2018-07-13

**Authors:** Valentina Sabatini, Valentina Pifferi, Stefano Checchia, Sara Rebeccani, Hermes Farina, Marco Aldo Ortenzi, Luigi Falciola

**Affiliations:** 1 Dipartimento di Chimica, Università degli Studi di Milano, Via Golgi 19, 20133 Milano, Italy; valentina.pifferi@unimi.it (V.P.); sara.rebeccani@studenti.unimi.it (S.R.); hermes.farina@unimi.it (H.F.); marco.ortenzi@unimi.it (M.A.O.); luigi.falciola@unimi.it (L.F.); 2 CRC Materials & Polymers Laboratory (LaMPo), Università degli Studi di Milano, Via Golgi 19, 20133 Milano, Italy; 3 Consorzio Interuniversitario per la Scienza e Tecnologia dei Materiali (INSTM), Via Giusti 9, 50121 Firenze, Italy; 4 European Synchrotron Radiation Facility (ESRF), 71 Avenue des Martyrs, 38000 Grenoble, France; stefano.checchia@esrf.fr

**Keywords:** conducting polymer, poly (3,4-ethylenedioxythiophene), sulfonated polyarylethersulfone, doping agent, XRD analyses, solvatochromism, modified electrode, casting solvent effect

## Abstract

Despite the poor solubility in organic solvents, poly (3,4-ethylenedioxythiophene) (PEDOT) is one of the most successful conducting polymers. To improve PEDOT conductivity, the dopants commonly used are molecules/polymers carrying sulfonic functionalities. In addition to these species, sulfonated polyarylethersulfone (SPAES), obtained via homogeneous synthesis with different degrees of sulfonation (DS), can be used thanks to both the tight control over the DS and the charge separation present in SPAES structure. Here, PEDOTs having enhanced solubility in the chosen reaction solvents (*N*,*N*-dimethylformamide, dimethylacetamide, dimethyl sulfoxide, and *N*-methyl-2-pyrrolidone) were synthesized via a high-concentration solvent-based emulsion polymerization with very low amounts of SPAES as dopant (1% *w*/*w* with respect to EDOT monomer), characterized by different DS. The influence of solvents and of the adopted doping agent was studied on PEDOT_SPAESs analyzing (i) the chemical structure, comparing via X-ray diffraction (XRD) the crystalline structures of undoped and commercial PEDOTs with PEDOT_SPAES’ amorphous structure; (ii) solvatochromic behavior, observing UV absorption wavelength variation as solvents and SPAES’ DS change; and (iii) electrochemical properties: voltammetric peak heights of PEDOT_SPAES cast onto glassy carbon electrodes differ for each solvent and in general are better than the ones obtained for neat SPAES, PEDOTs, and glassy carbon.

## 1. Introduction

In recent years, the fabrication of conductive electrodes based on conductive polymer composites (CPCs) has gained an increasing academic and industrial research interest. Moreover, the use of conductive coatings has been growing for many different applications in smart fields, such as sensors, photovoltaics, and portable and flexible electronics; some examples of smart devices include pollutant detectors for industrial and civil waters, touchscreens displays, organic and inorganic solar cells, and capacitors [1].The filler network in the bulk polymer plays an important role in the electrical conductivity of CPCs [2,3,4] and, up to now, metal nanoparticles [5], organic metal complexes, and hybrids [6] have been widely used to prepare conductive electrodes, but their industrial application is limited by several disadvantages, such as poor processability and high costs [7]. Among them, silver and titanium-based electrodes are currently the most promising metal electrodes and are under rapid development for applications in smart electronic devices. Even if different silver and titanium coatings have been formulated and tested, the high cost and the low metal content limit their widespread industrial applications [8].

Many research groups have fabricated conductive electrodes using different carbon-based fillers, such as carbon nanotubes (CNT) [9], graphene (G) [10] and graphene oxide (GO) [11]. Though all these materials offer excellent conductive properties, the raw materials are expensive and not easily modifiable in order to improve the conductive features of the final devices [12]. Thus, there is a need to develop new types of conductive electrodes able to overcome the technical problems previously reported for metals and carbon-based coatings and, accordingly, there are pressing needs to develop large-scale, low-cost fabrication routes of high-performance conductive electrodes.

Several alternatives to metal nanoparticles, organic metal complexes, hybrids, CNTs, G, and GO, such as conducting polymers, have been developed: in fact, the synthesis and, therefore, the properties of a conducting polymer can be appropriately designed and tailored to meet the requirements for electrochemical applications In this context, poly (3,4-ethylenedioxythiophene) (PEDOT) is considered the most promising candidate to be used to manufacture highly flexible and printable electrodes due to a number of advantages, i.e., simplicity of manufacture, good mechanical stability, and high optical transmittance [13]. On the other hand, although PEDOT possesses good electrical conductivity [14], it is insoluble in many common organic solvents, making it difficult to be processed [15].

2-Naphthalenesulfonic acid (Naph), an organic molecule with one sulfonic moiety, is, in general, introduced in PEDOT formulations as a template to synthesize PEDOT_Naph water solutions with improved solubility in organic solvents, currently representing one of the most successful commercial products based on conductive polymers [16]. PEDOT_Naph water solutions with different conductivities have been successfully used in many devices, including polymer light-emitting diodes, conductive inks, capacitors, and transistors. Naph works as a template and dopant in a PEDOT_Naph dispersion, and favors water solubility of PEDOT. However, the insulating properties of Naph raise some issues because they make it difficult to balance the conductivity and processing ability of PEDOT_Naph dispersion. A high Naph-to-PEDOT ratio results in good stability of the dispersion and film formation, but decreases conductivity of the film coating itself. It is desirable to enhance the useful properties of PEDOT and its derivatives in conductive patterns and, thus, the key to the success is to develop new types of PEDOT electrodes to solve the above-mentioned problems.

The present study aims to fill this technological gap by reporting, for the first time, the preparation of electrodes based on highly conductive and processable PEDOT coatings. For the above considerations, PEDOTs characterized by improved solubility in the chosen reaction solvents, i.e., *N*,*N*-dimethylformamide (DMF), dimethylacetamide (DMAc), dimethyl sulfoxide (DMSO), and *N*-methyl-2-pyrrolidone (NMP) were successfully synthesized via a high-concentration solvent-based emulsion polymerization with ferric sulfate as the radical initiator. Furthermore, the oxidative polycondensation of 3,4-ethylenedioxythiophene (EDOT) monomer in the reaction solvents reported above and in the presence of a new kind of doping agent, sulfonated polyarylethersulfone (SPAES), that was synthesized on purpose, is illustrated.

SPAES has received great attention in the last decade due to the possibility to use it for a variety of separation processes, e.g., ion exchange membranes, reverse osmosis, and electrodialysis processes [17]. It is a completely amorphous polymer, characterized by excellent UV and thermal resistance (e.g., high glass transition temperature, *T*_g_, up to 310 °C and low thermal expansion coefficient), optical properties, chemical stability, oxidation resistance, as well as by good mechanical properties and easy processability [18]. In our previous work [19], SPAES membranes were tested and compared with Nafion^®^ (Dupont^TM^, Wilmington, DE, USA), as electrode modifier for applications in electroanalysis. Different parameters, such as polymer acid moieties’ counter-ion (acidic or alkaline salt), solvent casting conditions, method of drying, and storage and influence on the performances of the membrane were evaluated. The research is still in progress and the ultimate goal should be the production of ad hoc tailored membranes, designed for the specific type of analyte and application under investigation.

SPAES can be prepared via two different synthetic routes; one is the synthesis of polyarylethersulfone followed by the post-sulfonation reaction with sulfonating agents, commonly defined as heterogeneous synthesis [20], while the other uses pre-sulfonated monomers in the feed of the polymerization reaction and is defined homogeneous synthesis [21]. In the present work, a series of SPAESs with increasing amounts of sulfonic groups—0.5, 0.75, and 1.0 meq R–SO_3_^−^·g^−1^ of polymer—was synthesized via homogeneous synthesis, i.e., the polycondensation reaction using a pre-sulfonated co-monomer, 2,5-dihydroxybenzene-1-sulfonate potassium salt. Thanks to SPAES’ homogenous synthesis, which allows the tight control over the DS, and to the presence of a charge separation in SPAES macromolecular structure due to the use of the pre-sulfonated co-monomer, SPAES was successfully tested as a PEDOT doping agent.

A low amount of SPAES dopant was used in the feed of the polymerization (1% *w*/*w* with respect to EDOT) and its influence and DS variation were studied on PEDOT_SPAES’s chemical structure, comparing via X-ray diffraction (XRD) measurements the crystalline structures of undoped PEDOT and PEDOT_Naph, synthesized as references, with PEDOT_SPAESs amorphous structure. Furthermore, the effect of the four reaction solvents and chosen doping agent was observed on PEDOT_SPAESs solvatochromic behavior: a UV absorption wavelength variation is clearly detectable as the reaction solvent’s polarity changes, a bathochromic shift in the case of DMF, DMAc, and NMP, and a hypsochromic shift for DMSO and SPAES DS increases. Additionally, the electroanalytical properties of PEDOT_SPAESs cast onto glassy carbon electrodes were investigated: it was found that the choice of the reaction solvent significantly influences the electrochemical behavior of the resulting electrode and, in the case of NMP PEDOT_SPAESs, voltammetric peak heights are, in general, better than the ones obtained for neat SPAESs, PEDOT, PEDOT_Naph, and glassy carbon.

To the authors’ best knowledge, this is the first time that SPAES was used as a dopant for PEDOT-based electrodes characterized by improved solubility in common organic solvents, i.e., DMF, DMAc, DMSO, and NMP, and that a study, although preliminary, of SPAES and solvents’ effects on PEDOT_SPAES’s structural properties, solvatochromic behavior and electroanalytical performances was performed. Thanks to PEDOT_SPAES’s solvatochromic and electroanalytical properties a new kind of conducting polymer for electrodes and sensors could be available for several research fields.

## 2. Materials and Methods

### 2.1. Materials

3,4-Ethylenedioxythiophene (EDOT, >97%, stored at +5 °C), 2-naphthalenesulfonic acid (Naph, >70%), ferric sulfate (Fe_2_(SO_4_)_3_·xH_2_O, >97%), 4,4′-difluorodiphenylsulfone (BFPS, ≥99%), and 4,4′–dihydroxydiphenyl (BHP, ≥97%) were supplied by Sigma Aldrich (Milan, Italy) and used without further purification and EDOT purity was checked via FT-IR and ^1^H NMR spectra [22]. 2,5-Dihydroxybenzene-1-sulfonate potassium salt (sulfonated hydroquinone, SHQ, ≥98%) was obtained from Alfa Aesar (Karlsruhe, Germany) and potassium carbonate (K_2_CO_3_, ≥98% anhydrous) was purchased from Fluka (Milan, Italy). *N*,*N*-dimethylformamide (DMF, 99.8% anhydrous), dimethylacetamide (DMAc, ≥99.5%), dimethyl sulfoxide (DMSO, ≥99.5%), *N*-methyl-2-pyrrolidone (NMP, ≥99.5% anhydrous), toluene (99.8% anhydrous), hydrochloric acid (1.00 M HCl), potassium chloride (0.1 M KCl), hexaammineruthenium (III) chloride ([Ru(NH_3_)_6_]Cl_3_, 98%), distilled water Chromasolv^®^ (≥99.9%), and dimethyl sulfoxide-d_6_ (DMSO-d_6_, 99.96 atom% D) were supplied by Sigma Aldrich and used without purification.

### 2.2. Synthesis of Poly (3,4-Ethylenedioxythiophene) (PEDOT) and PEDOT Doped with 2-Naphthalenesulfonic Acid (PEDOT_Naph)

Four PEDOTs obtained using different reaction solvents, namely, DMF, DMAc, DMSO, and NMP, were synthesized. In a representative polymerization procedure, a 50 cm^3^ glass test tube equipped with magnetic stirring is loaded with EDOT (2.50 g), Fe_2_(SO_4_)_3_·xH_2_O (5 g) as a radical initiator and reaction solvent (3 cm^3^). The polymerization mixture is stirred for 24 h at room temperature and under an air atmosphere. The obtained solutions (brown in the case of DMF, orange for DMAc, green for DMSO, and purple in the case of NMP), i.e., the polymerization products, are analyzed without purification. Furthermore, four PEDOT_Naph samples were synthesized as described in the case of PEDOT samples, adding Naph (0.025 g, 1% *w*/*w* with respect to EDOT monomer) as a doping agent.

### 2.3. Synthesis of Sulfonated Polyarylethersulfones (SPAESs)

Three SPAESs with increasing nominal degree of sulfonation (DS), expressed as meq R–SO_3_^−^·g^−1^ of polymer—SPAES_0.5, SPAES_0.75, SPAES_1—were synthesized. The exact amount of the monomers used for the syntheses are reported in Table 1. In a representative polymerization procedure, BFPS, BP, SHQ, and K_2_CO_3_—the latter used as a proton scavenger—are introduced into a 100 cm^3^ one neck round-bottom flask equipped with a modified Dean-Stark device, with magnetic stirring and under a nitrogen atmosphere. The flask, loaded with toluene and NMP in order to have a 10% *w*/*v* concentration of the monomers in the solution is put in an oil bath and the polymerization reaction is carried out under reflux for 6 h; the water formed during the reaction is removed as an azeotrope with toluene through the modified Dean-Stark device. After complete water removal, the polymerization temperature is gradually increased to 198 °C and then the reaction mixture is kept for 18 h at 198 °C. The dark-purple polymerization mixture obtained is precipitated into a large excess of cold water under magnetic stirring and a brown solid is obtained. The polymerization product is recovered via filtration and washed with cold water from residual monomers, solvents, and K_2_CO_3_. The difficult removal of NMP and toluene from SPAESs required repeating the purification procedure several times. After drying in a vacuum oven (about 4 mbar) at 200 °C for 24 h, the presence of residual solvents was checked via isothermal TGA analysis (2 h at 220 °C under nitrogen flow). Therefore, SPAESs were converted to the acid form by immersion of each sample in 50 cm^3^ of 1 M HCl solution for 24 h at 100 °C, followed by washing with water for 24 h at 25 °C and drying in a vacuum oven (about 4 mbar) at 150 °C for 24 h.

### 2.4. Synthesis of PEDOT Doped with SPAES (PEDOT_SPAES)

The four syntheses of PEDOT polymers, as described previously in Section 2.2, were repeated introducing SPAES as the doping agent. For each solvent, the polymerization reaction was performed changing SPAES DS—0.5/0.75/1.0 meq R–SO_3_^−^·g^−1^ of polymer. PEDOT_SPAES samples were synthesized as described in the case of PEDOTs, adding SPAES (0.025 g) at 1% *w*/*w* with respect to EDOT monomer.

### 2.5. SPAES, PEDOT, PEDOT_Naph, and PEDOT_SPAES Characterization

#### 2.5.1. Nuclear Magnetic Resonance: ^1^H NMR

**Figure 1:**
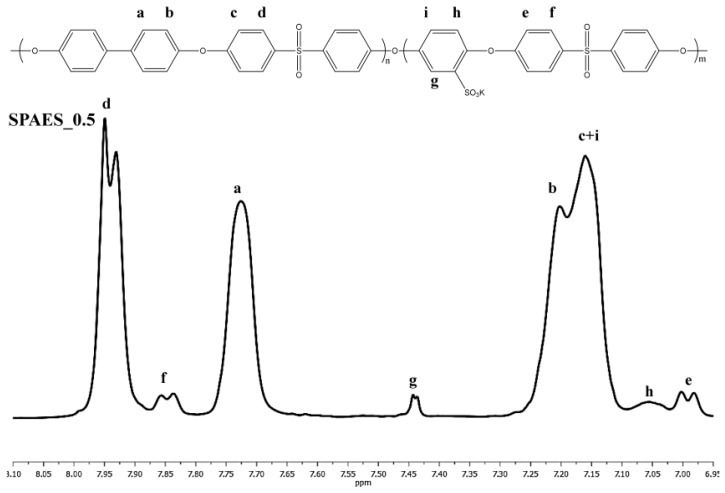
^1^H NMR of SPAES_0.5.

^1^H NMR spectra were collected at 25 °C with a BRUKER 400 MHz spectrometer (Milan, Italy). Samples for the analyses were prepared dissolving 8–10 mg of polymer in 1 cm^3^ of DMSO-d_6_. The presence of R–SO_3_^−^ groups in SPAES samples was quantitatively measured via ^1^H NMR spectroscopy calculating the integral ratios between the proton in ortho to the sulfonic group (g) of SHQ and the ones of BFPS (d), (f) and of BHP (a), as reported in Figure 1 for ^1^H NMR spectrum of SPAES_0.5, using Equation (1) (where I_g_ is the integral area of peak (g); I_d,f_ is the integral area of the peaks (d) and (f); *I*_a_ is sthe integral area of the peak (a); Ur_BFPS_ is the molecular weight of the BFPS repeat unit, i.e*.* 216.25 g·mol^−1^; Ur_BP_ is the molecular weight of the BP repeat unit, i.e. 184.21 g·mol^−1^; and Ur_SHQ_ is the molecular weight of the SHQ repeat unit, i.e. 226.26 g·mol^−1^).

DS = ((*I*_g_ × 1000))/(((*I*_d,f_ × Ur_BFPS_)/4) + ((*I*_a_ × Ur_BP_)/4) + (*I*_g_ × Ur_SHQ_)) (1)

#### 2.5.2. Potentiometric Titration (PT)

The presence of R–SO_3_^−^ groups in SPAES samples in terms of ion exchange capacity (IEC) was also determined via potentiometric titration. A solution of benzoic acid (60 mg in 50 cm^3^ of water) was prepared. A 0.01 M NaOH solution was used for titration. A titration of pure water using the same NaOH solution (75 cm^3^ of water) was performed first, and the NaOH solution volume used for the titration is indicated as VH_2_O (cm^3^) [23]; then, 10 cm^3^ of benzoic acid solution was titrated, obtaining the NaOH solution volume used for the neutralization, *V*_benz_ (cm^3^). The solvent factor (*f*) was calculated using Equation (2):

*f* = *m*/(*V*_titr_ × *M*_benz_ × [NaOH]) (2)

where m is the weight of benzoic acid in solution (g); *V*_titr_ is given by the difference between *V*_benz_ and VH_2_O (cm^3^); [NaOH] is the concentration of 0.01 M NaOH solution (mol·cm^−3^) and M_benz_ is the molecular weight of benzoic acid (122.12 g·mol^−1^). SPAESs IEC was measured after soaking the protonated sample in 20 cm^3^ of 2 M NaCl solution for at least 24 h. The solution was then titrated with 0.01 M NaOH using a Titrino751 GPD automatic potentiometric titrator (Metrohm, Herisau, Switzerland). IEC data can be calculated with Equation (3) (where [NaOH] is the 0.01 M NaOH solution; *V* is the NaOH solution volume used during the neutralization of each sample; *f* is the solvent factor obtained as described in Equation (2); x is the weight of the sample (g).

IEC = ([NaOH] × *V* × *f*)/x (3)

#### 2.5.3. Intrinsic Viscosity (η)

SPAESs samples’ intrinsic viscosity was measured using an Ubbelohde viscometer in a thermostatic water bath at 25 °C. Samples were dissolved in DMAc and analyzed in the concentration range of 1 to 0.3 g·dL^−1^.

#### 2.5.4. Size Exclusion Chromatography (SEC)

SPAESs molecular weight was evaluated via SEC analyses using a Waters 1515 Isocratic HPLC pump (Milan, Italy), three Waters Styragel columns set (HR3-HR4-HR5) and a refractive index (RI) detector Waters 2487. SEC analyses were performed at 25 °C, with a flow rate of 1 cm^3^·min^−1^ and 40 μL as injection volume. Samples were prepared by dissolving 40 mg of polymer in 1 cm^3^ of anhydrous DMF and then filtered with 0.45 μm filters. The calibration was built using the following monodispersed Polystyrene (PS) standards: *M*_p_ = 1,600,000 Da (*D* ≤ 1.13), *M*p = 1,150,000 Da (*D* ≤ 1.09), *M*_p_ = 900,000 Da (*D* ≤ 1.06), *M*_p_ = 400,000 Da (*D* ≤ 1.06), *M*_p_ = 200,000 Da (*D* ≤ 1.05), *M*_p_ = 90,000 Da (*D* ≤ 1.04), *M*_p_ = 50,400 Da (*D* = 1.03), *M*_p_ = 30,000 Da (*D* = 1.06), *M*_p_ = 17,800 Da (*D* = 1.03), *M*_p_ = 9730 Da (*D* = 1.03), *M*_p_ = 5460 Da (*D* = 1.03), *M*_p_ = 2032 Da (*D* = 1.06), *M*_p_ = 1241 Da (*D* = 1.07), *M*_p_ = 906 Da (*D* = 1.12), *M*_p_ = 478 Da (*D* = 1.22); Ethyl benzene (molecular weight = 106 g·mol^−1^). For all analyses, 1,2-dichlorobenzene was used as internal reference.

#### 2.5.5. Differential Scanning Calorimetry (DSC)

DSC analyses were conducted using a Mettler Toledo DSC 1 (Columbus, DE, USA), under nitrogen atmosphere on samples of SPAESs, PEDOTs, PEDOT_Naphs and PEDOT_SPAESs weighing from 5 to 10 mg each. The temperature program for SPAESs is as follows: (i) heating from 25 to 330 °C at 10 °C·min^−1^; (ii) 5 min of isotherm at 330 °C; (iii) cooling from 330 to 25 °C at 10 °C·min^−1^; (iv) 5 min isotherm at 25 °C; and (v) heating from 25 to 330 °C at 10 °C·min^−1^ (*T*_g_ was measured here). After drying in a vacuum oven (about 4 mbar) at 200 °C for 24 h, PEDOTs, PEDOT_Naphs, and PEDOT_SPAESs were analyzed with the following thermal program: (i) heating from 25 to 250 °C at 10 °C·min^−1^; (ii) 5 min of isotherm at 250 °C; (iii) cooling from 250 to 25 °C at 10 °C·min^−1^; (iv) 5 min isotherm at 25 °C; and (v) heating from 25 to 480 °C at 10 °C·min^−1^ (as reported for SPAES samples, *T*_g_ was measured here).

#### 2.5.6. Thermogravimetric Analyses (TGA)

TGA analyses were performed using a TGA 4000 Perkin Elmer instrument (Waltham, MA, USA), under a nitrogen atmosphere, on samples weighing from 5 to 10 mg each and heating from 30 to 800 °C at 10 °C·min^−1^. PEDOT, PEDOT_Naph and PEDOT_SPAES samples were analyzed after drying in a vacuum oven (about 4 mbar) at 200 °C for 24 h.

#### 2.5.7. UV-Visible Spectrophotometry

A Shimadzu UV-160 recording double-beam UV–visible spectrophotometer with a data processing system (Milan, Italy) was used. UV–visible spectra of sample solutions were recorded in 1 cm quartz cells at a scan speed of 40 nm·min^−1^. The absorbance and absorption maximum wavelength of PEDOTs, PEDOT_Naphs, PEDOT_SPAESs and SPAESs in their solutions of DMF, DMAc, DMSO, and NMP (concentration of 0.1% *w*/*w*) were determined between in wavelength ranges of 1100–190 nm. Moreover, the absorbance and absorption maximum wavelength of DMF, DMAc, DMSO, and NMP solvents were measured in water solutions at 0.1% *w*/*w* of concentration.

#### 2.5.8. X-Ray Diffraction (XRD) Characterization

**Figure A1:**
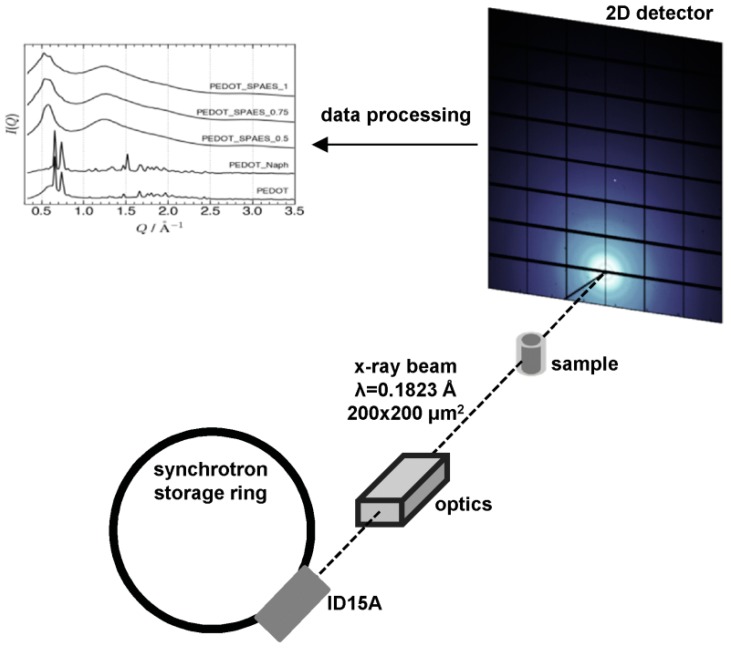
Schematic of the high-energy X-ray diffraction setup. Data processing to obtain one-dimensional data is outlined in the Experimental section of the main manuscript.

**Figure A2:**
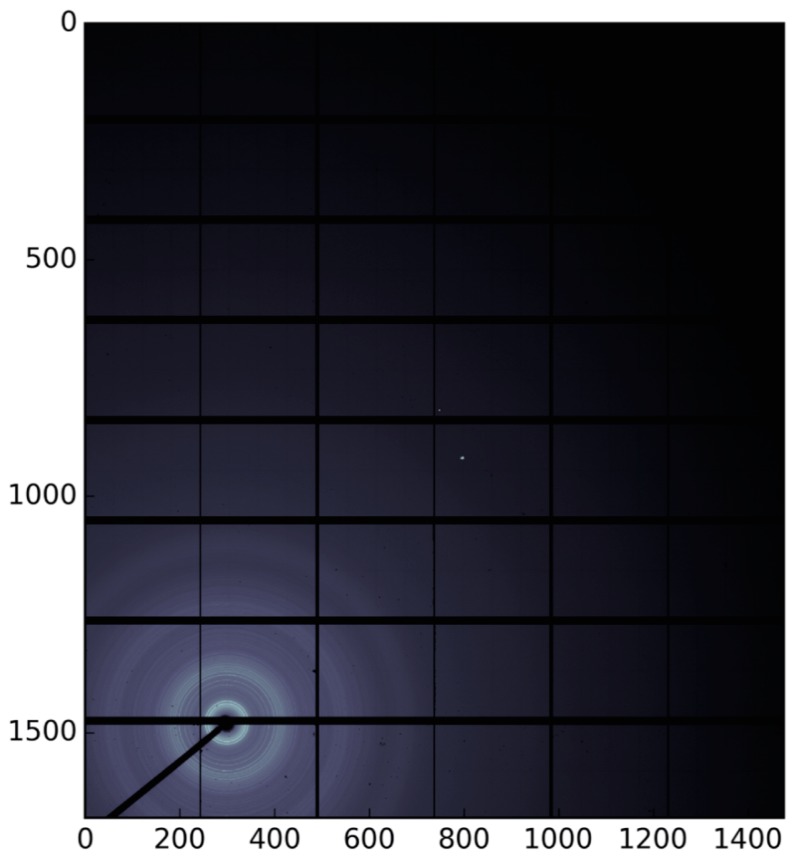
Raw two-dimensional images of PEDOT.

**Figure A3:**
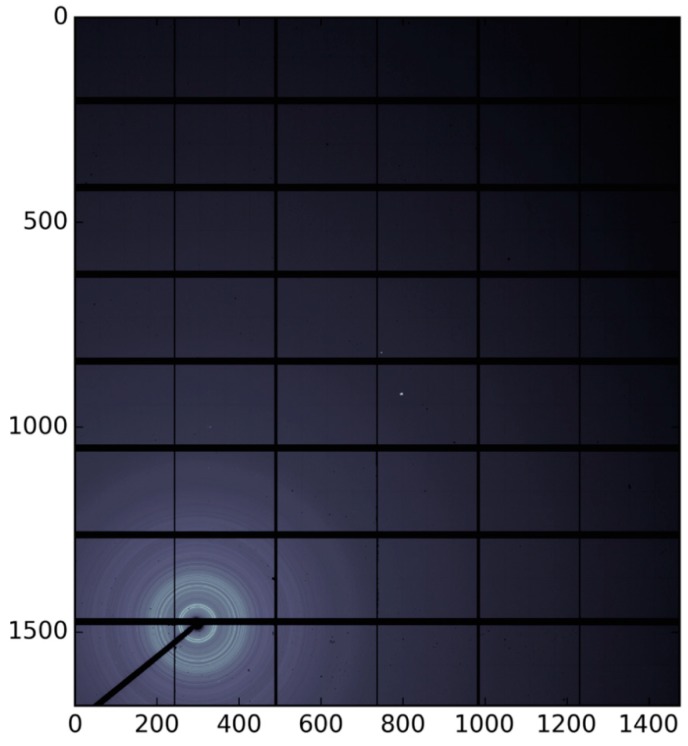
Raw two-dimensional images of PEDOT_Naph.

**Figure A4:**
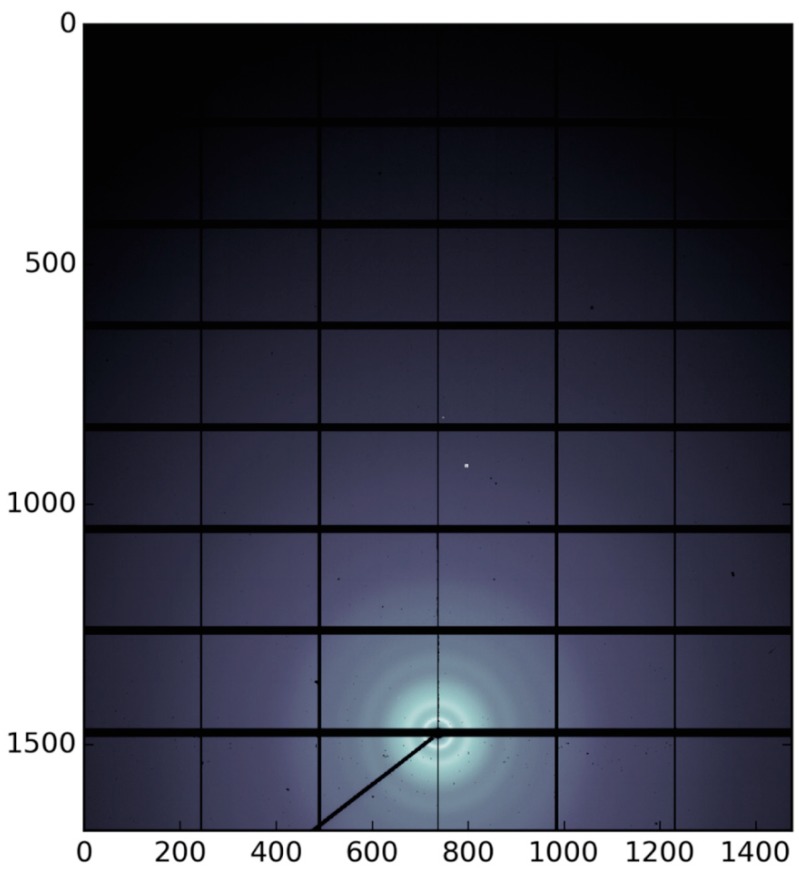
Raw two-dimensional images of PEDOT_SPAES_0.5.

**Figure A5:**
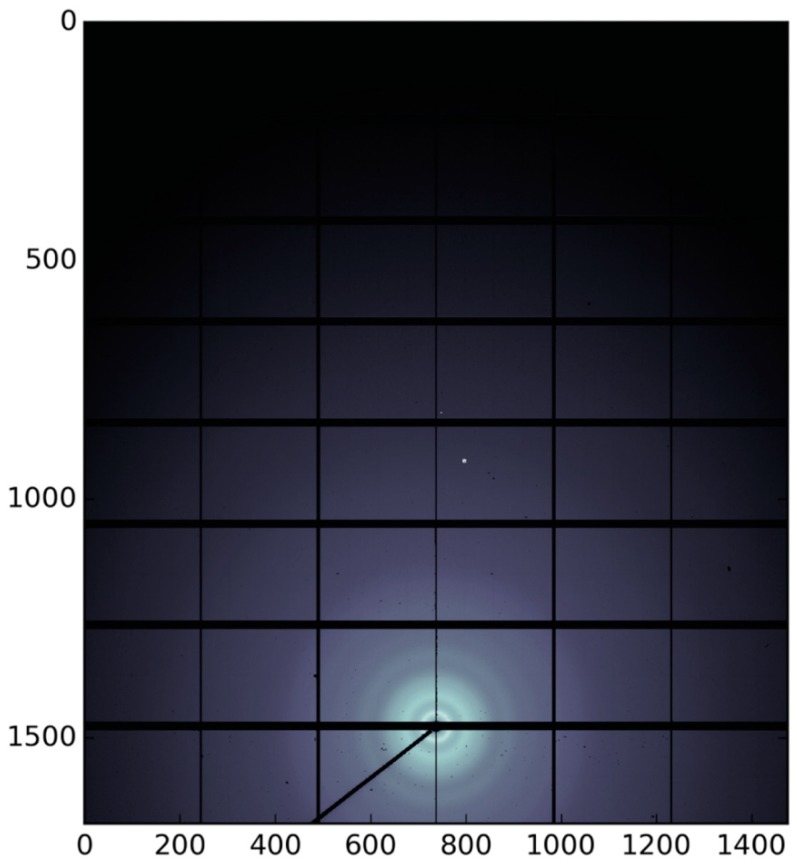
Raw two-dimensional images of PEDOT_SPAES_0.75.

**Figure A6:**
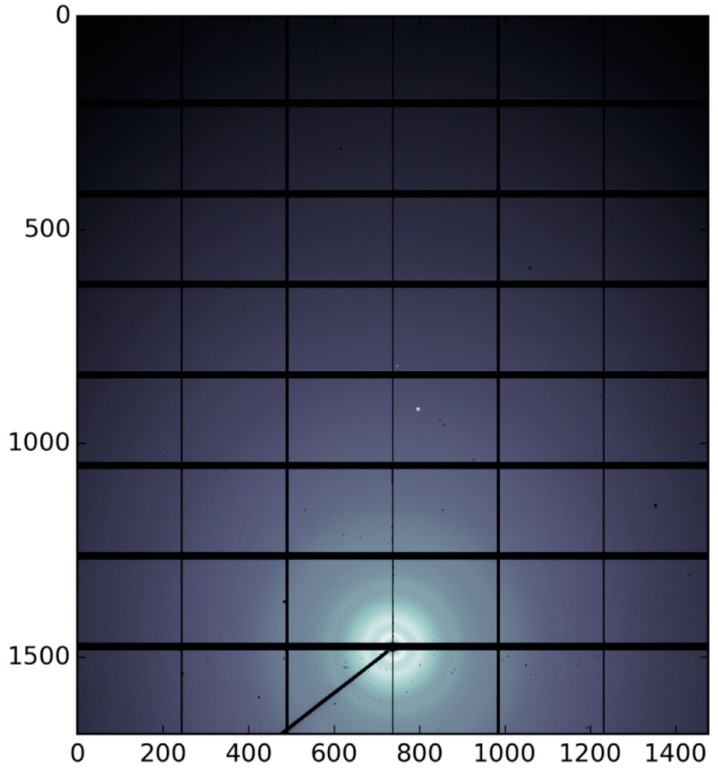
Raw two-dimensional images of PEDOT_SPAES_1.

Diffraction patterns of undoped PEDOT, PEDOT_Naph, and three PEDOT_SPAES samples (PEDOT_SPAES_0.5, PEDOT_SPAES_0.75, and PEDOT_SPAES_1) were collected at the beamline ID15A at the ESRF synchrotron (Grenoble, France). 1 mm-thick rods of solution-cast polymer were mounted on a goniometric head in the center of a rotation stage (Aerotech) and measured in transmission geometry with a 200 × 200 μm^2^ beam. X-ray energy was 68 keV (wavelength = 0.18233 Å), allowing measurements in a range of momentum transfer (*Q*) up to *Q*_max_ = 20.0 Å^−1^. Diffraction images were recorded with a Dectris Pilatus 2 M CdTe during a total exposure time of 120 s per sample. A schematic representation of the high-energy X-ray diffraction setup used is reported in Figure A1 (Appendix A). All the samples showed broad homogeneous diffraction rings with no sign of preferred orientation. Raw two-dimensional images of the five samples analyzed are reported in Figure A2, Figure A3, Figure A4, Figure A5 and Figure A6 (Appendix A). The intensities of the Debye-Scherrer rings in the two crystalline samples (PEDOT, PEDOT_Naph) are evenly distributed over all azimuthal angles. Detector calibration for the five datasets took into account the shift in the detector horizontal position. Raw images were scaled by the incident photon flux, averaged, and subtracted by the background scattering using the Python library FabIO [24]; azimuthal integration was done using pyFAI [25]; the atomic pair distribution function (PDF) was obtained as the *G*(r) function using PDFgetX3 [26]. PDF analysis of diffraction data is a method to study the real-space atomic structure in liquids and non-crystalline solids through the Fourier transform of the total scattering intensity. Peaks in the PDF correspond to the probability of finding pairs of atoms at a distance r, weighted by the atomic number and the molar fraction of the elements in the pair [27].

#### 2.5.9. Cyclic Voltammetric Characterization

Cyclic voltammetric experiments were performed with an Autolab PG-Stat204 potentiostat/galvanostat (Ecochemie, The Netherlands) in a conventional electrochemical cell, by using saturated calomel, a Pt wire, and a polymer-modified glassy carbon electrode as the reference, counter, and working electrodes, respectively. The potential was scanned from +0.3 V to −0.7 V with a scan rate of 0.1 V·s^−1^ and a step potential of 0.005 V. The electrochemical probe is 3 mM [Ru(NH_3_)_6_]Cl_3_ in 0.1 M KCl as the supporting electrolyte. Prior to the modification, the glassy carbon electrode (3 mm of diameter) was polished with synthetic diamond powder (diameter 1 mm) on a Struers DP Nap wet cloth and rinsed in water. A solution 0.5 *w*/*w* of the chosen polymer was prepared with the selected solvent and 20 µL were deposited on the glassy carbon surface. The electrode was dried under controlled temperature (30, 40, or 50 °C) and pressure (250 mbar).

## 3. Results and Discussion

### 3.1. Synthesis and Characterization of PEDOTs, PEDOT_Naphs, SPAESs and PEDOT_SPAESs

PEDOT is usually synthesized via oxidative polymerization of EDOT monomer either in the absence of solvent or in the presence of water. Both routes yield polymers that are poorly soluble in common organic solvents and, therefore, are difficult to process [28]. Moreover, PEDOT polymerization is conducted in the presence of an agent carrying sulfonic moieties used both as a stabilizer and a dopant for the polymer itself. During EDOT oxidative polymerization, the formation of the cationic charged PEDOT occurs, strongly interacting with the R-SO_3_^−^ group of the doping agent and forming a stable PEDOT/dopant complex. Examples of commercial sulfonated PEDOT dopants are either organic molecules, for example, 2-naphtalene sulfonic acid (Naph) and para toluene sulfonic acid, or sulfonated polymers, i.e., sulfonated polystyrene (PSS) [29].

**Figure 2:**
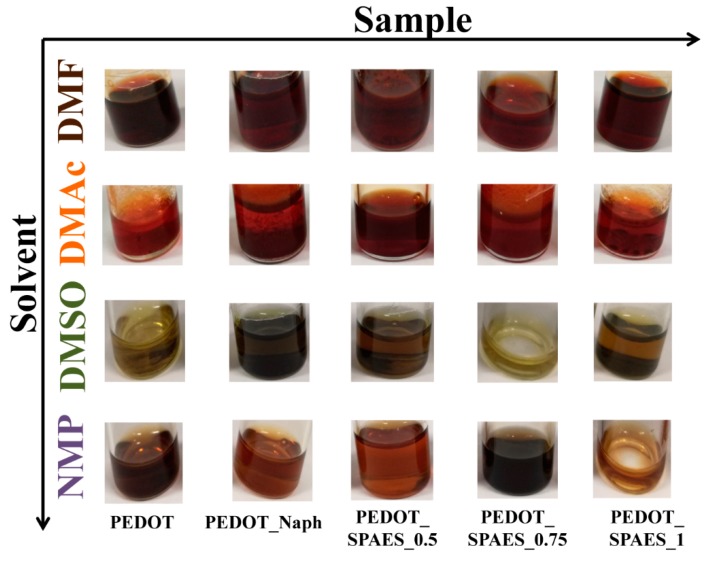
Summary of the polymeric solutions synthesized and their appearance.

In this work, PEDOT and PEDOT_Naph were obtained via a direct oxidative polycondensation reaction of EDOT monomer, in the presence of an oxidant species (ferric sulfate) and in four different solvents (DMF, DMAc, DMSO, and NMP). PEDOT_Naph was doped with 1% *w*/*w* of Naph, having a nominal DS of 4.8 meq R–SO_3_^−^·g^−1^ of polymer. The four reaction solvents were chosen in order to solubilize the new sulfonic doping agent tested in this work, SPAES, which is soluble in DMF, DMAc, DMSO, and NMP. The polymeric solutions obtained are completely soluble in all four solvents and appear in different colors as shown in Figure 2. All samples were dried in a vacuum oven and the overall compositions were measured via ^1^H NMR spectra [22].

In addition to the reported PEDOT dopants, SPAES can also be used as a PEDOT doping agent thanks to the possibility to appropriately modulate the number of sulfonic groups and to tailor R–SO_3_^−^ groups’ distribution homogeneity along the polymeric chains. To the best of the authors’ knowledge, no use of SPAES in the field of PEDOT dopants has been reported yet.

**Figure 3:**
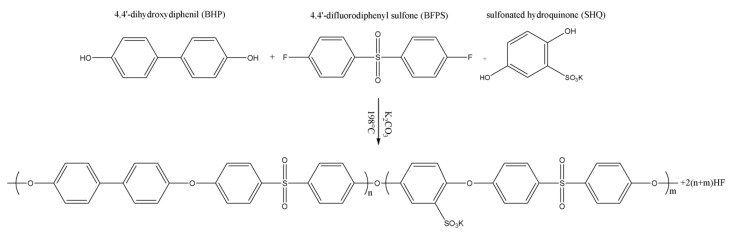
Synthesis route for SPAESs.

In view of the above considerations, SPAESs with three nominal DS, 0.5–0.75–1.0 meq R–SO_3_^−^·g^−1^ of polymer, were synthesized as described in Section 2.3; Figure 3 reports the representative procedure for the homogeneous synthesis of SPAESs by direct co-polymerization of BFPS and BHP with a sulfonated monomer, SHQ; the full description of SPAESs homogeneous synthesis and their macromolecular characterizations are reported in our previous work [21].

Table 2 shows the macromolecular and thermal properties of SPAESs: DS (calculated via ^1^H NMR spectroscopy), IEC (measured via potentiometric titration), molecular weights determined by intrinsic viscosity (η) and size exclusion chromatography (SEC) analyses, and *T*_g_ data measured via differential scanning calorimetry (DSC). These results indicate that SHQ monomer successfully reacted in all the samples synthesized. The attainment of high molecular weights is detectable from the comparison of viscosity and SEC data between commercial PES (Radel^®^ A-A-300A, Solvay, Bruxelles, Belgium) [30] and SPAESs samples prepared. SPAESs synthesized have very high *T*_g_ values that increase as the DS increases: SPAES_0.5 *T*_g_ is 260 °C, a value that increases up to 291 °C for SPAES_0.75 and to 304 °C for SPAES_1.

In view of the high molecular weights, high *T*_g_ data and the charge separation due to the presence of the sulfonated co-monomer (SHQ), SPAES was used here as a doping agent for PEDOT polymers. PEDOT_SPAES samples were obtained via a direct oxidative polycondensation reaction of EDOT monomer, in the presence of (i) ferric sulfate as a radical initiator; (ii) SPAES with increasing DS –0.5/0.75/1 meq R–SO_3_^−^·g^−1^ of polymer as a dopant; and (iii) a reaction solvent chosen among DMF, DMAc, DMSO, and NMP. All PEDOT_SPAES polymeric solutions obtained have excellent solubility in each reaction solvent and appear brown in the case of DMF, orange for DMAc, green for DMSO, and purple in the of NMP, as shown in Figure 2. After drying in a vacuum oven (about 4 mbar) at 200 °C for 24 h, the thermal stability of PEDOT_SPAESs samples was estimated via thermogravimetric analyses (TGA) evaluating temperatures corresponding to 1%, 5%, and 10% of weight loss (*T*_1%_, *T*_5%_, and *T*_10%_) and comparing results obtained with TGA data of PEDOTs and PEDOT_Naphs used as references (Table 3). DSC and TGA thermograms corresponding to the data presented are reported in according with our previous work [22].

PEDOT is rather stable up to the temperature of 270 °C. From 270 °C a continuous degradation occurs until major decomposition appears in the region between 300 and 350 °C, owing to the thermal degradation of thiophene rings [31]. Two steps of weight loss characterize the typical TGA curve of SPAES: thermal decomposition of the R–SO_3_^−^ groups at around 300–330 °C and the thermal degradation of the polymer starting from around 450–500 °C, related to the breaking of –SO_2_^−^ bonds and the degradation of the polymeric chains respectively [21].

Associating *T*_1%_ with the breaking of thiophene bonds, *T*_5%_ with the thermal decomposition of R–SO_3_^−^ groups and *T*_10%_ with both the breaking of –SO_2_^−^ bonds and the degradation of thiophene rings, it can be clearly seen from Table 3 that *T*_1%_, *T*_5%_ and *T*_10%_ of PEDOT_SPAESs are much higher than the ones of PEDOT and PEDOT_Naph. Comparing the data trends of PEDOT_SPAESs, thermal stability significantly increases as the DS of SPAES increases. In general, PEDOT_SPAESs seem to be more stable up to 300 °C (*T*_1%_) thanks to the bulky anionic structure of SPAES. Starting from 300 °C, a continuous degradation occurs with the thermal decomposition of R-SO_3_^−^ groups (*T*_5%_) and the degradation of SPAES polymeric chains (*T*_10%_).

In addition, T_g_ values of PEDOT, PEDOT_Naph and PEDOT_SPAES samples was measured via DSC. As reported in Table 3, PEDOT_SPAESs T_g_ is slightly higher than undoped PEDOTs. This is probably due to the increased amount of R–SO_3_^−^ groups introduced into the polymer chains; furthermore, PEDOT_Naphs synthesized have comparable *T*_g_ respect to PEDOT_SPAESs. No significant correlation between reaction solvent and PEDOT_SPAESs thermal stability was detected.

### 3.2. X-Ray Diffraction (XRD) Characterization

**Figure 4:**
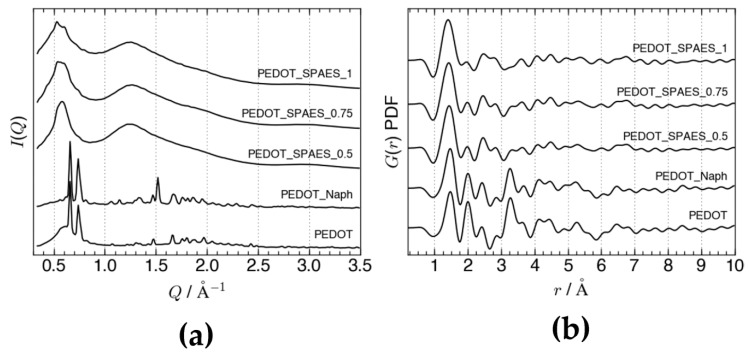
(**a**) Low-angle region of the diffraction patterns of the five samples as a function of momentum transfer (Q). (**b**) Pair distribution function curves of the same samples up to 10 Å; the first peak around *r* = 1.40 Å contains all the nearest-neighbor bond distances in the polymers. The five datasets are offset for clarity in both panels.

The different effect of the two doping agents compared in this work, Naph and SPAES, on the structural order of PEDOT was evidenced by XRD measurements. While Naph does not disrupt the long-range order of undoped PEDOT, doping with amorphous SPAES [32,33], despite the low loading of SPAES used (1% *w*/*w* with respect to EDOT monomer), results in amorphous phases and more structural disorder on the bond-length scale. The integrated diffraction patterns of the samples PEDOT, PEDOT_Naph, PEDOT_SPAES_0.5, PEDOT_SPAES_0.75, and PEDOT_SPAES_1 are plotted in Figure 4a; the corresponding PDF curves can be seen in Figure 4b.

Undoped PEDOT and PEDOT_Naph appear as nanocrystalline, with the main Bragg peaks at *Q* = 0.66 Å^−1^ and *Q* = 0.74 Å^−1^ corresponding to d-spacings of 9.52 Å and 8.49 Å, respectively. The I (*Q*) patterns of all three SPAES-doped samples, instead, consist of three broad features whose d-spacings are still related to nanocrystalline PEDOT, in line with the diffraction patterns reported for undoped PEDOT thin films and PEDOT doped with Naph [16], tosylate, poly (4-styrenesulfonate), and PSS [34,35,36,37,38]. The first amorphous peak spans the range 0.51 < *Q* < 0.63 Å^−1^ and can be attributed to a broad distribution of side-by-side interchain spacings as in the orthorhombic phase identified by Aasmundveit et al. [34]. Side-by-side spacings separate PEDOT layers stacked along the a-axis and alternated with layers of dopant molecules arranged parallel to the bc plane. In this case, the SPAES dopant induces a fine structure in the first peak, with two main d-spacings at *d* = 11.9 Å and *d* = 10.5 Å (*d* = 2π/Q) emerging when DS is increased over 0.5 meq R–SO_3_^−^·g^−1^ of polymer. The formation of the preferred interlayer spacings at high DS can be attributed to stronger coulombic interactions between the sulfonic acid moieties of SPAES and the polar C–O bonds in thiophene, constraining the conformation of SPAES chains between two adjacent PEDOT layers. The significant shift towards the low-Q of the main features in PEDOT_SPAESs reflects the larger steric hindrance of SPAES compared with the Naph-based dopant, in light of both the free rotation allowed between neighboring aromatic rings in the former and the impossibility of the latter to stack vertically in the interstices between PEDOT layers.

The layered structure in PEDOT_SPAES samples is also reflected by the second amorphous peak around *Q* = 1.25 Å^−1^, which corresponds to half an interlayer spacing. The d-spacings related to PEDOT layers stacked face-to-face, finally, only appear as a shoulder at *Q* = 1.80 Å^−1^ (*d* = 3.4 Å), while the peak appearing around *Q* = 0.9 Å^−1^ (*d* = 6.8 Å) in PEDOT/tosylate [34] is absent in all the samples. Ordered stacking of PEDOT chains, however, is still visible in the short-range structure of undoped PEDOT, whose PDF curve has peaks at *r* = 3.4 and *r* = 6.8 Å (Figure 4b). These two peaks are decreased only slightly by Naph, but are much weaker in PEDOT_SPAESs. Moreover, increasing the DS in the SPAES dopant leads to the disappearance of the distance at *r* = 3.4. The drop in intensity at *r* = 3.4 Å points to the misalignment of nearest-neighboring PEDOT chains and shows that SPAES doping affects the structure of PEDOT down to the bond-length scale.

### 3.3. UV–Visible Spectrophotometry Characterization: A Study of Solvatochromic Behavior of PEDOTs, PEDOT_Naphs, PEDOT_SPAESs, and SPAESs Polymeric Solutions

Solvatochromism is the capability of a chemical substance to change color as the polarity of a solvent changes. Varying the difference in dipole moment between the ground and excited states of the chromophore, two different kinds of solvatochromism are possible: a negative solvatochromism corresponds to hypsochromic shift, i.e., blue shift, with increasing solvent polarity; on the other hand, the corresponding bathochromic shift, i.e., red shift, is named positive solvatochromism [39].

The solvatochromic shift of a chromophore reflects a strong correlation between the absorption and emission spectra with solvent polarity. Since polarities of the ground and excited state of a chromophore differ, a variation in the solvent polarity will lead to different stabilization of the ground and excited states and, therefore, a change in the energy gap between these two electronics states. Variations in the position, intensity, and shape of the absorption spectra can be direct measures of solute and solvent molecule interactions [40]. Due to the Frank-Condon principle, the excited state solvent shell is not in equilibrium with the excited state molecule, i.e., the solute [41].

From Figure 2, it is evident that the polymeric solutions synthesized in this work, changing the reaction solvent, appear with different colors as solvent polarity changes: brown in the case of DMF (polarity index of 40.4), orange for DMAc (polarity index of 40.1), green for DMSO (polarity index of 44.4), and purple in the case of NMP (polarity index of 36) [42].

**Figure 5:**
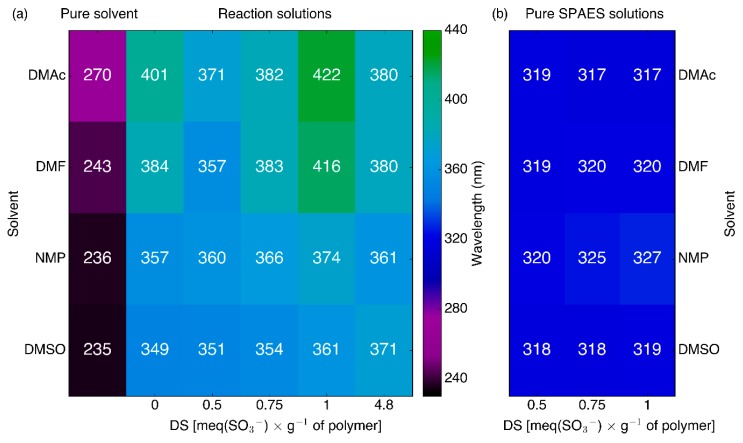
Wavelengths of the absorption maxima of (**a**) pure DMAc, DMF, NMP, and DMSO, and the polymeric solutions of undoped PEDOT, PEDOT_Naph (DS = 4.8), and PEDOT_SPAES (DS = 0.5–0.75–1); and (**b**) pure SPAES dissolved in the same solvents. The same color scale is used in both plots.

**Figure A7:**
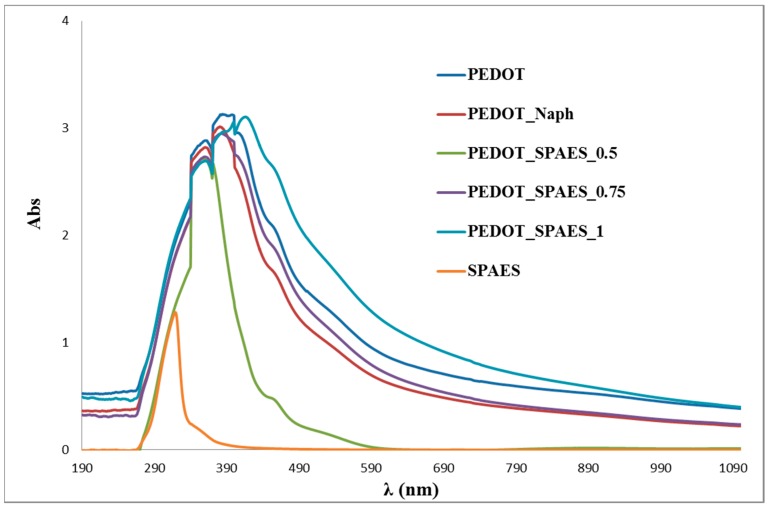
UV absorbance data of PEDOT, PEDOT_Naph, PEDOT_SPAES, and SPAES samples synthesized in DMF.

**Figure A8:**
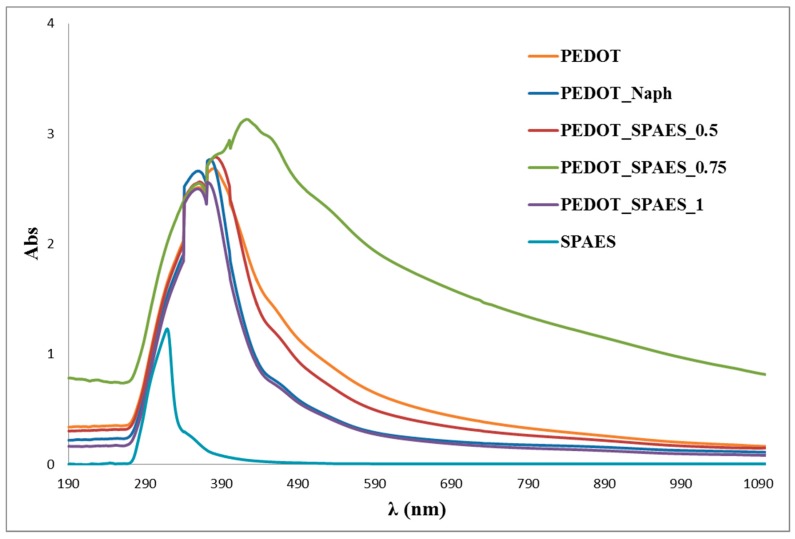
UV absorbance data of PEDOT, PEDOT_Naph, PEDOT_SPAES, and SPAES samples synthesized in DMAc.

**Figure A9:**
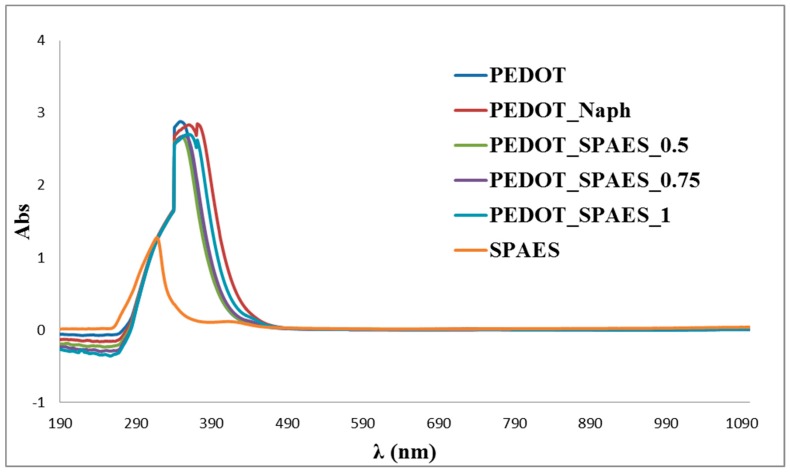
UV absorbance data of PEDOT, PEDOT_Naph, PEDOT_SPAES, and SPAES samples synthesized in DMSO.

**Figure A10:**
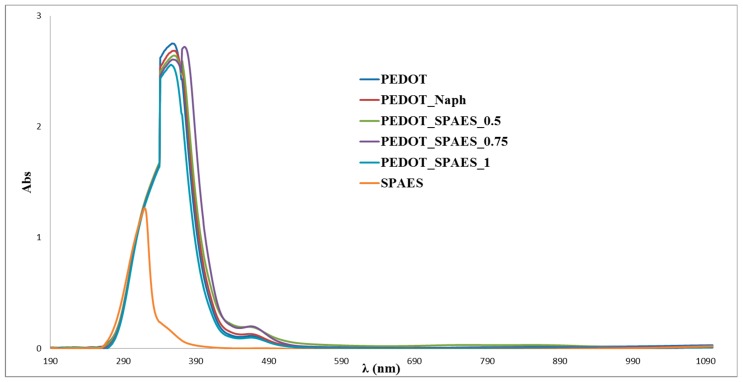
UV absorbance data of PEDOT, PEDOT_Naph, PEDOT_SPAES, and SPAES samples synthesized in NMP.

To investigate the solvatochromic properties of PEDOTs, PEDOT_Naphs and PEDOT_SPAESs polymeric solutions, all samples were analyzed via UV absorption measurements (UV absorbance data corresponding to the data presented here are reported in Appendix A, Figure A7, Figure A8, Figure A9 and Figure A10). Figure 5a shows the absorption maximum wavelengths of PEDOTs, PEDOT_Naphs, and PEDOT_SPAESs polymeric solutions in the reaction solvents DMF, DMAc, DMSO, and NMP. The first column of Figure 5a reports the absorption maximum wavelengths of the pure solvents.

Correlating the wavelength of maximum absorption with the reaction solvent used for PEDOTs, PEDOT_Naphs, and PEDOT_SPAESs, the absorption maxima of all samples fall at the shortest wavelength in the case of DMSO, the solvent with the highest polarity index (44.4). A bathochromic shift with respect to the DMSO series appears evident for all the samples synthesized in solvents with a lower polarity index: NMP (36), DMAc (40.1), and DMF (40.4).

Comparing the series of PEDOT_SPAESs and PEDOT_Naphs samples synthesized in the same reaction solvent, it can be noted that the absorption maximum wavelength (i) increases with the DS of SPAES, and (ii) is less sensitive to solvent choice in PEDOT_Naph solutions. To determine whether the solvatochromic behavior of PEDOT_SPAES comes from the SPAES dopant or is unique to doped PEDOT polymers, the UV spectra of pure SPAES samples were measured after solubilization in the same solvents (Figure 5b). SPAES, regardless of its DS, has the same absorption maximum wavelength value in all the solvents within 8 nm. SPAES is not a solvatochromic polymer, but its chemical interaction with PEDOT as a doping agent influences PEDOT solvatochromic behavior. Therefore, the unique solvatochromic properties of PEDOT_SPAESs could be attractive for sensing applications.

### 3.4. Cyclic Voltammetric Characterization

In our previous works [22,43], PEDOT_SPAES thin films were tested for their conductivity, as conductive inks for organic electronics. The research conducted leads to modified PEDOT polymers with conductivity of 210 S·cm^−1^, 50 S·cm^−1^ higher than the one of commercial PEDOT. The research is still in progress and, up to now, the goal of this work is the production of ad hoc tailored electrodes for electrochemical investigations.

**Figure 6:**
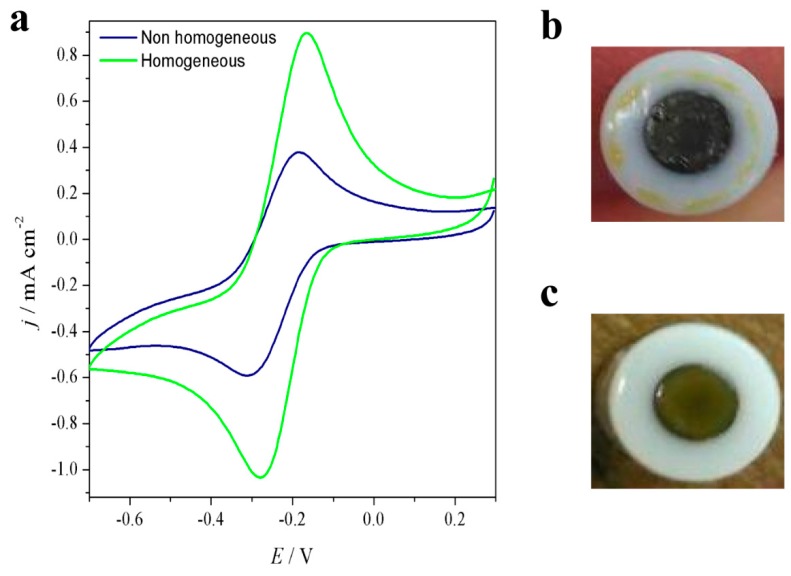
(**a**) Cyclic voltammograms of PEDOT_SPAES_1 (i) homogeneous casting conditions: 50 °C, 250 mbar, 140 min of drying, and (ii) non homogeneous casting conditions: 50 °C, 250 mbar, 390 min; (**b**) non-homogenous PEDOT_SPAES_1; and (**c**) homogeneous PEDOT_SPAES_1. The electrochemical probe is 3 mM [Ru(NH_3_)_6_]Cl_3_.

Preliminary cyclic voltammetric experiments were performed using an electrochemical probe molecule with positive charges, [Ru(NH_3_)_6_]Cl_3_, able to interact with the negatively-charged moieties of the SPAES doping agent [19]. The goal of this study was to identify the best casting conditions in terms of temperature, pressure, and time of drying of the polymeric solutions cast onto the surface of glassy carbon electrodes in order to detect the effect of SPAES dopant and SPAES DS in PEDOT polymers compared to neat PEDOT and a commercial PEDOT doping agent, i.e., Naph. The homogeneity of the cast polymeric film (i.e., formation of a regular polymeric layer without any bubbles or cracks and firmly attached onto the surface of the glassy carbon electrode) is a key factor to obtain high peak currents. As shown in Figure 6a for PEDOT_SPAES_1 dissolved in NMP, an inhomogeneous film largely degrades the peak currents. A suboptimal deposition like the one shown in Figure 6c (compared with an optimal one in Figure 6b) occurs when the drying time is too long and is not balanced by a matching change in temperature and pressure.

To find the ideal casting parameters, films of PEDOTs, PEDOT_Naphs, and PEDOT_SPAESs prepared from polymeric solutions in the four reaction solvents (DMF, DMAc, DMSO, and NMP) were cast and dried onto the surface of glassy carbon electrodes in a range of temperature, pressure, and time of drying, and the heights of their voltammetric peaks compared. Table A1, Table A2, Table A3 and Table A4 list all of the tests conducted.

Previous results about SPAES [19] demonstrated the strict dependence of the electrochemical performances of the polymeric film on the casting solvent. For the materials described here, this dependence is taken to extremes since, for some solvents, no stable films can be obtained.

The worst case in terms of film stability is DMSO (Table A3), where all the tested samples drop off the glassy carbon surface after immersion in the supporting electrolyte. In subsequent works, the morphology of DMSO-based films cast on glassy carbon surfaces will be investigated in order to understand the reason of this behavior.

**Figure 7:**
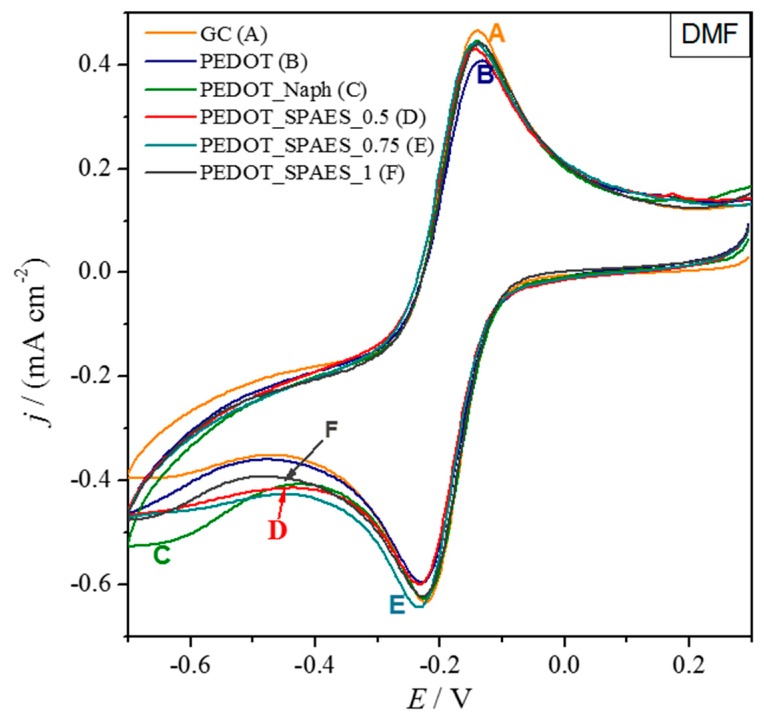
Cyclic voltammograms of bare glassy carbon (GC) and modified GC with polymers dissolved in DMF (Table A1). The electrochemical probe is 3 mM [Ru(NH_3_)_6_]Cl_3_.

**Figure A11:**
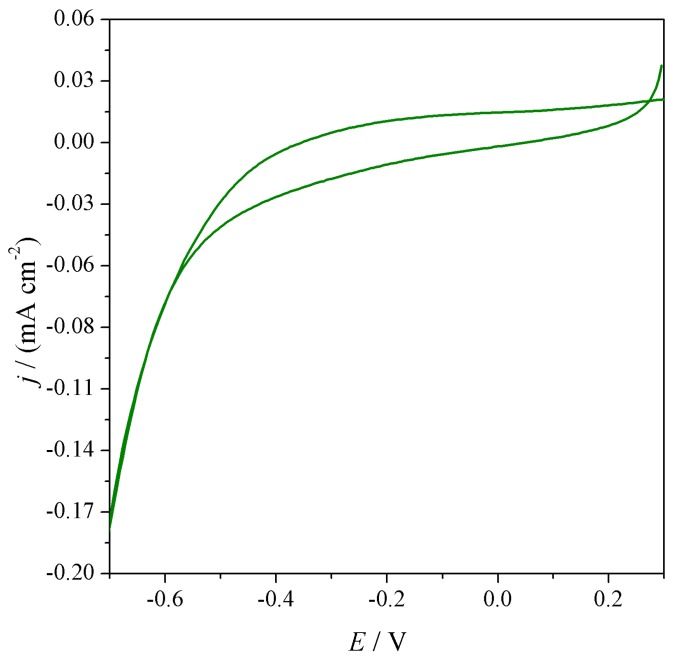
Cyclic voltammogram of PEDOT dried from DMF in KCl 0.1 M.

When DMF is the casting solvent (Table A2), all the tested films are stable, but give an electrochemical response very similar to bare glassy carbon (Figure 7). In accordance with our previous investigations, this effect is attributed to the short drying time of the low-boiling DMF, which prevents the formation of a high active area, and a good 3D architecture [19]. Furthermore, the cyclic voltammograms of PEDOT dried from DMF in KCl 0.1 M without the additional redox system is reported in Appendix A, Figure A11.

**Figure 8:**
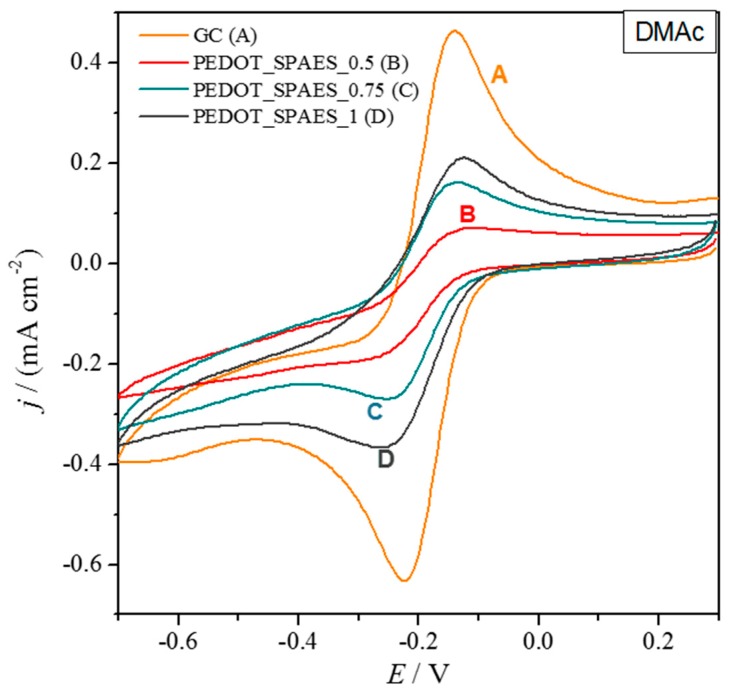
Cyclic voltammograms of bare glassy carbon (GC) and modified GC with polymers dissolved in DMAc (Table A2). The electrochemical probe is 3 mM [Ru(NH_3_)_6_]Cl_3_.

In the case of DMAc (Table A2), stable samples are obtained only when SPAES is used as doping agent, whereas PEDOT and PEDOT_Naph are impossible to test. At any DS the electrochemical signal of PEDOT_SPAES is lower than that of glassy carbon (Figure 8) and is directly dependent on the amount of sulfonic groups. Film stability shows the same trend, probably concurring with the slight increase in peak current.

**Figure 9:**
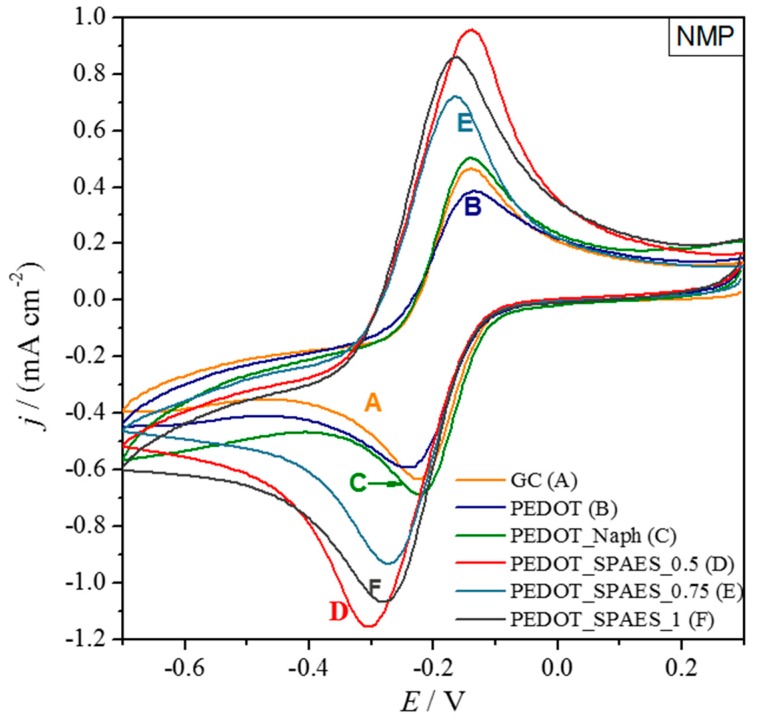
Cyclic voltammograms of bare glassy carbon (GC) and modified GC with polymers dissolved in NMP (Table A4). The electrochemical probe is 3 mM [Ru(NH_3_)_6_]Cl_3_.

Finally, NMP-cast films of PEDOT_SPAES gave the best electrochemical performances (Table A4), in all cases better than glassy carbon. The best electrode, at DS = 0.5 meq R–SO_3_^−^·g^−1^ of polymer, showed peak currents double those of glassy carbon (Figure 9). The high boiling point of NMP allows the best organization of the 3D film structure, giving higher active area [23].

**Figure A12:**
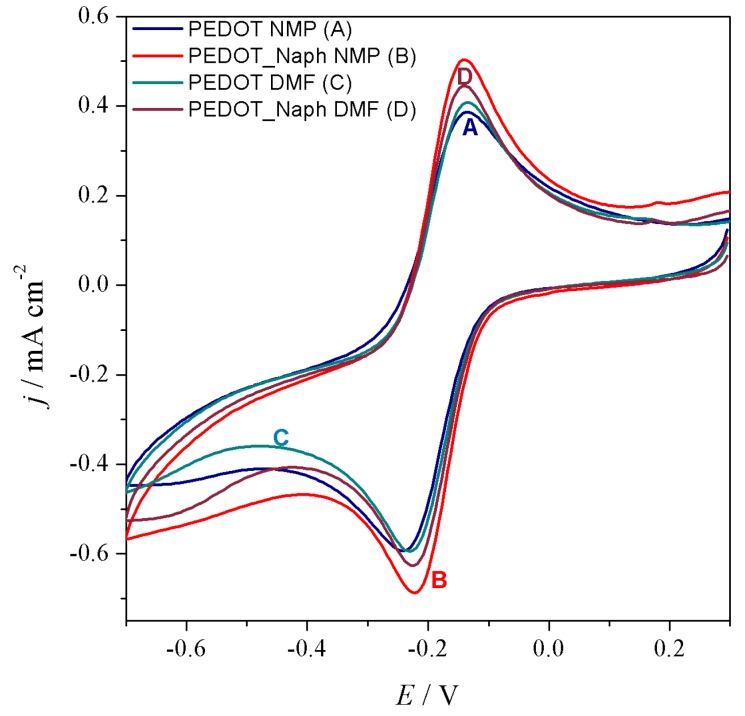
Cyclic voltammograms of PEDOT and PEDOT_Naph dissolved in DMF or NMP (Table A1 and Table A4). The electrochemical probe is 3 mM [Ru(NH_3_)_6_]Cl_3_.

Moreover, it is important to consider the two blank samples, PEDOT and PEDOT_Naph (cast from DMF and NMP), whose voltammograms are reported in Figure A12. Their electrochemical signal is very similar independent of the solvent. More important, PEDOT_Naphs contain significantly more sulfonic groups (4.8 meq R–SO_3_^−^·g^−1^ of polymer) than any of the PEDOT_SPAESs, but unlike PEDOT_SPAESs they fail to improve the electrochemical performance of PEDOT. The marked increase in peak currents, the solvatochromic effect, and the structural changes evidenced by XRD demonstrate that the doping with SPAES completely modifies the electrochemical response of PEDOT, which becomes solvent-dependent and exploits better than the Naph dopant in the presence of sulfonic groups. In further studies SPAES as a doping agent for PEDOT polymers will be investigated with other electrochemical probes.

## 4. Conclusions

To overcome the well-known technical problems of poly (3,4-ethylenedioxythiophene) (PEDOT), i.e., difficult processability and patterning, due to its poor solubility in common organic and inorganic solvents, PEDOTs characterized by a full miscibility in the reaction solvents used were successfully synthesized by a high-concentration solvent-based emulsion polymerization reaction between ethylenedioxythiophene (EDOT) and an oxidant species, i.e., ferric sulfate, in four different organic reaction solvents: *N*,*N*-dimethylformamide (DMF), dimethylacetamide (DMAc), dimethyl sulfoxide (DMSO), and *N*-methyl-2-pyrrolidone (NMP).

The oxidative polycondensation of EDOT in the presence of sulfonated polyarylethersulfone (SPAES) as a doping agent was characterized by three increasing degrees of sulfonation (DS), i.e., 0.5, 0.75 and 1.0 meq R–SO_3_^−^·g^−1^ of polymer, and was performed for the first time, leading to new conducting materials, PEDOT_SPAES polymers, characterized by full solubility in the reaction solvents chosen.

Despite the low amount of SPAES-based dopant used, only 1% *w*/*w* with respect to the EDOT monomer, its influence on the PEDOT_SPAES structure was studied via X-ray diffraction (XRD) spectra comparing unmodified PEDOT and PEDOT doped with 2-naphthalenesulfonic acid (PEDOT_Naph), having crystalline structures, with PEDOT_SPAESs, that are characterized by amorphous structures.

Both SPAES and the reaction solvents used influenced the solvatochromic behavior and the electrochemical features were assessed; a UV absorption wavelength variation is clearly detectable as the reaction solvents polarity change and SPAES DS increases. Moreover, the voltammetric properties of PEDOT_SPAESs cast onto glassy carbon electrodes were investigated: it was found that the choice of reaction solvent and the casting solvent conditions adopted significantly influences the electrochemical behavior of the resulting electrode and, in the case of NMP, PEDOT_SPAESs’ voltammetric peak heights, in general, are better than the ones obtained for neat SPAES, PEDOT, PEDOT_Naph, and glassy carbon.

Thanks to the unique properties deriving from the combined use of PEDOT with SPAES in different reaction solvents, a new kind of conducting polymers for electrodes and sensors could be available for several research fields. The work is continuing with an emphasis placed on the modification of SPAES macromolecular structure, i.e., from a linear structure to a branched one, with the subsequent study of its effect on PEDOT macromolecular and structural properties, and on the investigation of PEDOT_SPAES’ electrochemical properties with a lead-based probe.

## Figures and Tables

**Table 1:** Loading of the reagents for SPAESs with different DS.

Sample	Nominal DS (meq R–SO_3_^−^·g^−1^ of Polymer)	BFPS (g)	BHP (g)	SHQ (g)	K_2_CO_3_ (g)
SPAES_0.5	0.5	3.17	1.85	0.58	3.72
SPAES_0.75	0.75	3.15	1.69	0.76	3.70
SPAES_1	1.0	3.10	1.32	1.16	3.64

**Table 2:** List of SPAESs synthesized with their DS, IEC, intrinsic viscosity values (η), number average molecular weights ($\bar{Mn}$), weight average molecular weights ($\bar{Mw}$), molecular weight distribution (*D*), and *T*_g_ values. SEC data are expressed in PS equivalents.

Sample	Nominal DS (meq R–SO_3_^−^·g^−1^ of Polymer)	DS (meq R–SO_3_^−^·g^−1^ of Polymer)	IEC (meq R–SO_3_^−^·g^−1^ of Polymer)	[η] (dL·g^−1^)	$\bar{Mn}\;(\mathrm{Da})$	$\bar{Mw}\;(\mathrm{Da})$	*D*	*T*_g_ (°C)
Radel^®^A-A-300A	-	-	-	0.17	22,200	39,800	1.8	230.8
SPAES_0.5	0.5	0.48	0.49	0.34	23,700	43,500	1.8	259.7
SPAES_0.75	0.75	0.70	0.74	0.49	25,400	48,600	1.9	290.9
SPAES_1	1.0	0.98	0.95	0.72	26,700	52,900	2.0	303.6

**Table 3:** Degradation and *T*_g_ data of PEDOTs, PEDOT_Naphs, and PEDOT_SPAES samples.

Solvent	Sample	*T*_1%_ (°C)	*T*_5%_ (°C)	*T*_10%_ (°C)	*T*_g_ (°C)
DMF	PEDOT	270.1	300.5	315.5	n.d.
	PEDOT_Naph	271.8	300.4	313.8	n.d.
	PEDOT_SPAES_0.5	299.9	319.6	370.4	300.8
	PEDOT_SPAES_0.75	299.8	322.4	371.0	301.5
	PEDOT_SPAES_1.0	300.3	337.8	377.9	301.7
DMAc	PEDOT	270.1	300.5	315.5	n.d.
	PEDOT_Naph	271.9	299.8	312.5	n.d.
	PEDOT_SPAES_0.5	300.9	317.5	368.9	300.1
	PEDOT_SPAES_0.75	299.8	321.9	369.8	301.9
	PEDOT_SPAES_1.0	299.5	337.5	377.5	300.6
DMSO	PEDOT	267.9	299.4	312.1	n.d.
	PEDOT_Naph	271.4	300.0	313.7	n.d.
	PEDOT_SPAES_0.5	300.0	317.9	369.7	298.7
	PEDOT_SPAES_0.75	299.5	321.6	369.9	298.5
	PEDOT_SPAES_1.0	299.9	338.0	377.6	299.9
NMP	PEDOT	266.1	297.9	312.0	n.d.
	PEDOT_Naph	271.6	299.6	313.1	n.d.
	PEDOT_SPAES_0.5	300.2	317.8	369.8	300.8
	PEDOT_SPAES_0.75	299.8	321.5	369.5	300.6
	PEDOT_SPAES_1.0	299.7	337.4	377.8	300.7

## Appendix A

**Table A1:** Drying temperature, pressure, and time of PEDOTs’, PEDOT_Naphs’, and PEDOT_SPAESs’ polymeric solutions in DMF, and the stabilization time and current of the corresponding modified electrode.

Sample	Drying *T* (°C)	Drying *P* (mbar)	Drying Time (min)	Stabilization Time (min)	*i_p,r_* Ru (A·cm^−2^)
PEDOT	50	250	17	5	−0.00057499
PEDOT_Naph	50	250	15	3	−0.00060576
PEDOT_SPAES_0.5	30	250	40	2	−0.00056190
PEDOT_SPAES_0.75	30	250	40	6	−0.00061539
PEDOT_SPAES_1	30	250	40	8	−0.00061803

**Table A2:** Drying temperature, pressure, and time of PEDOTs’, PEDOT_Naphs’, and PEDOT_SPAESs’ polymeric solutions in DMAc, and the stabilization time and current of the corresponding modified electrode.

Sample	Drying *T* (°C)	Drying *P* (mbar)	Drying Time (min)	Stabilization Time (min)	*i_p,r_* Ru (A·cm^−2^)
PEDOT	30	250	195	0	n.d.
PEDOT_Naph	30	250	150	0	n.d.
PEDOT_SPAES_0.5	30	250	405	14	−0.00016187
PEDOT_SPAES_0.75	30	250	180	5	−0.00024393
PEDOT_SPAES_1	30	250	395	7	−0.00034997

**Table A3:** Drying temperature, pressure and time of PEDOTs’, PEDOT_Naphs’, and PEDOT_SPAESs’ polymeric solutions in DMSO, and the stabilization time and current of the corresponding modified electrode.

Sample	Drying *T* (°C)	Drying *P* (mbar)	Drying Time (min)	Stabilization Time (min)	*i_p,r_* Ru (A·cm^−2^)
PEDOT	30	250	10,035	n.d.	n.d.
PEDOT	40	250	2720	n.d.	n.d.
PEDOT	50	250	2755	n.d.	n.d.
PEDOT_SPAES_0.5	50	250	205	n.d.	n.d.
PEDOT_SPAES_1	30	250	10,035	n.d.	n.d.

**Table A4:** Drying temperature, pressure, and time of PEDOTs’, PEDOT_Naphs’, and PEDOT_SPAESs’ polymeric solutions in NMP, and the stabilization time and current of the corresponding modified electrode.

Sample	Drying T (°C)	Drying P (mbar)	Drying Time (min)	Stabilization Time (min)	*i_p,r_* Ru (A·cm^−2^)
PEDOT	30	250	8640	15	−0.00056039
PEDOT	40	250	604,320	0	n.d.
PEDOT	50	250	1740	0	n.d.
PEDOT_Naph	50	250	1740	10	−0.00064239
PEDOT_SPAES_0.5	40	250	270	15	−0.00113915
PEDOT_SPAES_0.75	40	250	180	13	−0.00092120
PEDOT_SPAES_1	40	250	270	8	−0.00102945
